# Poloxamer 407 and Hyaluronic Acid Thermosensitive Hydrogel-Encapsulated Ginsenoside Rg3 to Promote Skin Wound Healing

**DOI:** 10.3389/fbioe.2022.831007

**Published:** 2022-07-05

**Authors:** Xiaojuan Peng, Chuanbo Ding, Yingchun Zhao, Mingqian Hao, Wencong Liu, Min Yang, Fengyan Xiao, Yinan Zheng

**Affiliations:** ^1^ College of Chinese Medicinal Materials, Jilin Agricultural University, Changchun, China; ^2^ Jilin Agricultural Science and Technology University, Jilin, China

**Keywords:** ginsenoside Rg3, hydrogel, wound healing, autophagy, microbiota

## Abstract

Ginsenoside Rg3 has shown beneficial effects in various skin diseases. The current interest in designing and developing hydrogels for biomedical applications continues to grow, inspiring the further development of drug-loaded hydrogels for tissue repair and localized drug delivery. The aim of the present study was to develop an effective and safe hydrogel (Rg3-Gel), using ginsenoside Rg3, and we evaluated the wound-healing potential and therapeutic mechanism of Rg3-Gel. The results indicated that the optimized Rg3-Gel underwent discontinuous phase transition at low and high temperatures. Rg3-Gel also exhibited good network structures, swelling water retention capacity, sustainable release performance, and excellent biocompatibility. Subsequently, the good antibacterial and antioxidant properties of Rg3-Gel were confirmed by *in vitro* tests. In full-thickness skin defect wounded models, Rg3-Gel significantly accelerated the wound contraction, promoted epithelial and tissue regeneration, and promoted collagen deposition and angiogenesis. In addition, Rg3-Gel increased the expression of autophagy proteins by inhibiting the MAPK and NF-KB pathways *in vivo*. It simultaneously regulated host immunity by increasing the abundance of beneficial bacteria and the diversity of the wound surface flora. From these preliminary evaluations, it is possible to conclude that Rg3-Gel has excellent application potential in wound-healing drug delivery systems.

## Graphical Abstract

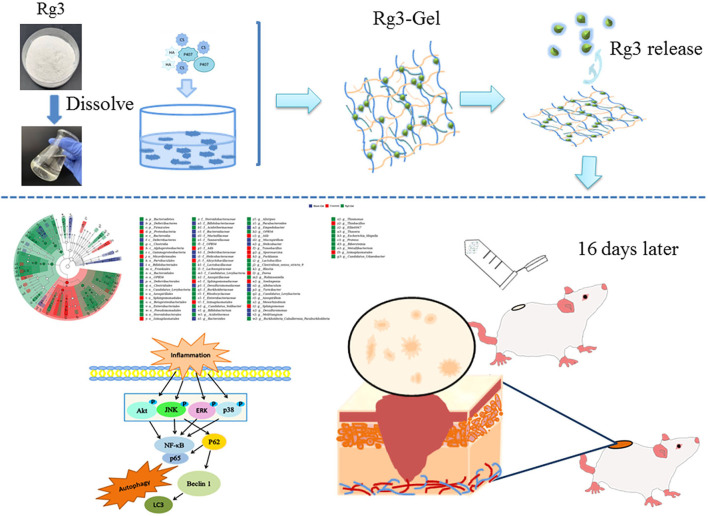

## 1 Introduction

The skin is the largest and most extensive organ in the human body. The skin acts as a wall between the organism and the exterior milieu to protect the underlying organs, perform physiological functions necessary for survival, and resist external pathogens given its constant exposure to potential injury in daily life (Proksch et al., 2008; Takeo et al., 2015). Wounds are among the most common skin conditions, representing a discontinuity and loss function of the epithelial integrity. High morbidity of cutaneous injuries imposes a significant burden on both the individual patient’s physical and healthcare economy, even requiring long-term painstaking treatment with the potential for cosmetic damage or death.

Wound healing, a complex coordinated process subjected to many intrinsic and exogenous imbalances, is an evolved dynamic biological process essential for species’ survival. Wound healing involves tightly controlled biochemical and cellular events, such as inflammation, proliferation, and remodeling, which occur sequentially in a continuous and sometimes overlapping fashion (Wang P.-H. et al., 2018; Oryan et al., 2019; Singer, 2022). The main goal of tissue regeneration in wound healing is the speed and quality of healing to effectively regenerate new healthy *epidermis* and lead to better cosmetic results. Wound management preparations should actively modulate the three phases of wound healing. A key concomitant activity in the proliferative phase of many healing wounds is the induced presence of vessel growth, a process known as angiogenesis (Veith et al., 2019). Epithelialization is another essential component of wound healing. Re-epithelialization begins with the migration of keratinocytes and participates in the cellular and molecular processes that initiate, maintain, and complete epithelialization, which in turn control wound closure and repair events (Pastar et al., 2014). During the proliferation and remodeling phases, various types of cells are extensively activated to induce the production of growth factors, following which, the wound surface recovers through re-epithelialization, collagen synthesis, matrix deposition, and vessel reconstruction (Reinke and Sorg, 2012; Pastar et al., 2014).

Emerging evidence has linked autophagy to skin wound healing. Recently, numerous studies have shown that autophagy is a process of cell catabolism to degrade and recover cytoplasmic components in eukaryotes (Sil et al., 2018; Ren et al., 2022). In addition to promoting the activation of inflammatory cells and enhancing their anti-inflammatory and anti-infective activities, autophagy is also beneficial to cell survival, migration, and proliferation associated with healing, thereby playing an important role in maintaining cell homeostasis under physiological and pathophysiological conditions. The impact of skin microbiome host interactions on wound-healing outcomes is another field of exciting research. The exact role of the microbiome in wound healing remains unclear, let alone the mechanisms of healing using therapeutics. Human skin harbors one billion microorganisms/cm2, collectively termed the microbiota, which can promote innate and adaptive immune responses to promote normal skin homeostasis and provide pathogen defense when the skin is intact (Pistone et al., 2021). Current studies suggest that many of these dimensions change, especially when wounds are formed, including microbial diversity, microbial load, and abundance of potential pathogens (Xu and Hsia, 2018). If the wound is not rapidly healed, the accumulation of inflammatory exudates can cause damage to the wound microenvironment and cell damage, following which, the skin microbes can multiply into sterile tissues, leading to bacterial overgrowth and infection, which thereby delay and complicate wound healing.

With an increased understanding of previously unknown cellular and molecular pathways involved in wound-healing processes, a broad range of further development is also underway based on new biomaterials and material fabrication techniques. So far, researchers are not only committed to rapidly expanding the range of research on wound dressings, such as sponges, foams, electrospun nanofibers, membranes, and hydrogels but also pay close attention to excavating their functions to exert advantage of their use in treatment (Dong and Guo, 2021). Hydrogels are generally synthesized from natural or synthetic crosslinked polymers and defined as highly hydrated three-dimensional (3D) linked network structures that have excellent ability to swell and bind several-fold more water or biological fluids, which enable them to be used as vehicles for the delivery of small and large drug molecules, providing them with versatile applications in biomedical areas (Tavakoli and Klar, 2020). Hydrogel networks can be cast into various sizes and shapes that are ideal to meet the demands of effective target drug delivery and rapid wound closure due to their unique properties. In particular, thermosensitive hydrogels are considered promising candidates for partial application of local therapy, which rapidly and reversibly undergo sol–gel transition behavior depending on ambient temperature changes, endowing the drug delivery capacity with controllable local spatial and temporal effects, as well as with moldability, biocompatibility, biodegradability, and tissue similarity to improve the bioavailability of drugs.

Ginsenoside Rg3 (Rg3) is one of the most important components from the traditional Chinese medicine Panax ginseng and has emerged as an effective anti-oxidative, anti-inflammatory, anticancer, antifatiguing, and cardioprotective medicine with evident effects (Kao et al., 2020). Recently, the use of Rg3 in the skin has started gaining considerable research attention, and Rg3 is considered to have therapeutic benefits in malignant melanoma, atopic dermatitis, skin senescence, and skin healing (Shan et al., 2014; Lee et al., 2018, 2019). Moreover, several studies have suggested that Rg3 serves as an early intervention for treating patients with hypertrophic scars (HS) by promoting wound healing in the early stage and inhibiting scar hyperplasia in the late stage (Cheng et al., 2013; Sun et al., 2014; Xu et al., 2019). Previous studies on the pharmacological effects of Rg3 can provide a theoretical basis for its application in skin treatment. However, Rg3 is a crystalline drug that is almost insoluble in water and dissolves only slightly in the mixed solvent of chloroform and methanol and dimethyl sulfoxide (DMSO) and other organic solvents; this greatly limits the bioavailability and formulation development of Rg3 (Yu et al., 2015). Therefore, the preparation of an effective drug delivery for Rg3 is critical to increase Rg3 solubility and improve absorption. In recent years, Rg3 has been tested in various formulations, including electrospun membranes, microparticles, microspheres, liposomes, and nanoparticles (Wang X. et al., 2018; Cheng et al., 2020). Some of the previous studies proposed the use of a nano-in-micro electrospun fiber membrane to fully exploit the potential of Rg3 as a skin repair drug delivery system (Cheng et al., 2020). However, to the best of our knowledge, no previous study has reported on the use of ginsenoside Rg3-loaded hydrogel wound dressings.

## 2 Materials and Methods

### 2.1 Materials

#### 2.1.1 Chemicals

Ginsenoside Rg3 was produced in the laboratory (the purified Rg3, having two configurations >95% total composition); P407 was supplied by BASF (Ludwigshafen, Germany); low-molecular-weight chitosan obtained from shrimp shells (*Pandalus borealis*) was purchased from Sigma-Aldrich (Shanghai) Trading Co., Ltd (the deacetylation degree of chitosan was 95.8%); sodium hyaluronate (15–25 MDa) was purchased from Shanghai Yuanye Bio-Technology Co., Ltd; SDS was purchased from Beijing Solarbio Science Technology Co., Ltd.; HPLC-grade acetonitrile (LiChrosolv^®^, CAS-No: 67-56-1) was purchased from Merck (Darmstadt, Germany); RIPA lysis buffer and BCA protein assay kit (BCA) were purchased from Beyotime Institute of Biotechnology (Jiangsu, China); the antibodies against p-ERK, p-JNK, and p-p38 were obtained from Cell Signaling Technology (Beverly, United States); antibodies against β-actin, GAPDH, and the goat antirabbit secondary antibody, NF-κB p65, Akt, ERK, JNK, p38, LC3, and Beclin-1 were obtained from the Proteintech (Santa Cruz, CA, United States); and antibodies against p-Akt and p62 were obtained from Arigo Biolaboratories Corp.

#### 2.1.2 Animal and Cell Lineages

Male ICR mice weighing 20–30 g were obtained from YiSi Experimental Animal Co., Ltd. (Chang Chun, China). The human immortalized keratinocytes (HaCat) cell line was purchased from Guangzhou Cellcook Biotech Co., Ltd.

DMEM medium, penicillin–streptomycin antibiotics (PS), fetal bovine serum albumin (FBS), and phosphate buffer saline (PBS) were purchased from Thermo Fisher Biochemical Products Co., Ltd. Moreover, 3-(4,5-Dimethyl-2-thiazolyl)-2,5-diphenyl-2H-tetrazolium bromide (MTT) was obtained from Aladdin Industrial Corporation (Shanghai, China).

### 2.2 Preparation of Rg3-Gel

The preparation of our hydrogel is partly based on the method explained by Soriano-Ruiza and colleagues (Soriano-Ruiz et al., 2020). In accordance with their published work, the required amounts of chitosan (CS) were prepared by adding the weight quantity in acetic acid solution (0.5% w/v) with continuous stirring until completely dissolved, and the required amounts of P407 were subsequently used for inclusion of Cs solutions using the cold method. HA 5% (w/v) solutions were prepared by adding hyaluronic acid (HA) to distilled water under continuous stirring for 1 h at room temperature and then utilized for inclusion of before adding Poloxamer 407(P407) and CS using the cold method. The precipitates of these polymer contents were then removed to get completely clear solutions obtained *in situ* forming hydrogels. Next, 10 mg Rg3 were pre-dissolved in 1 ml of SDS aqueous solution (0.4%, w/v) and then mixed with 9 ml of P407/Cs/HA in drug-loaded hydrogels using an AB mixing tube.

### 2.3 Characterization of Rg3-Gel

Fourier transform infrared spectroscopy (FT-IR) of the hydrogel was performed to confirm the chemical compositions of the prepared Rg3:P407:Cs:HA (Rg3-Gel) hydrogels. Both freeze-dried Rg3-Gel and Blank-Gel were mixed with KBr and compressed into a flake, the samples were scanned and recorded by FT-IR, and the spectroscopy was performed on a Thermo (United States) Nicolet iS50 in the wavelength range of 500–4,000 cm^−1^.

The X-ray diffraction (XRD) curves of the freeze-dried hydrogels and raw Rg3, P407, Cs, and HA were recorded using an X-ray diffractometer (JDX-3532 JEOL Japan), with the angle of diffraction (2θ) and the counts. The diffraction angle ranged from 10° to 80° at 45 kV and 40 mA.

The surface morphological structure of the prepared Rg3-Gel was examined by using a scanning electron micro-scope (SEM, BX-51; Olympus; Tokyo, Japan), and photographs were taken using a lens at ×800 and ×4000 magnification power. Before the observation, all the samples were freeze-dried and sputter-coated with gold.

### 2.4 Properties of Rg3-Gel

#### 2.4.1 Swelling Behavior

The swelling ratio of the Blank-Gel and Rg3-Gel were studied by the gravimetric method. The oven-dried samples of the hydrogels were weighed (Wd) and recorded before being immersed in phosphate-buffered saline (PBS) at 37°C. At regular intervals, the swollen samples were removed and the weight (Ww) was measured after carefully wiping off the surface moisture. The measurements were continued until the equilibrium swelling state was achieved.

The swelling ratio was calculated according to the following equation:

Swelling ratio(%)=(Ww-Wd)/ Wd,

where Ww and Wd represent the weight of the hydrogel after swelling and the weight of the hydrogel after freeze-drying, respectively.

#### 2.4.2 *In Vitro* Drug Release

The *in vitro* drug release experiment of Rg3-Gel was performed with slight modification according to a previously described method (Song et al., 2019). Briefly, hydrogel samples with PBS buffer solution (pH 7.4, 0.01 M) were placed into a 10-ml centrifuge tube at 37°C. Equal amounts of samples were collected at predetermined time intervals and added to the volume of fresh PBS buffer solution medium in order to maintain a simulated release condition. Then, the concentration of Rg3 was determined by using high performance liquid chromatography (HPLC) analysis (Waters 2,695). The mobile phase consisted of acetonitrile (solvent A), water (solvent B), and acetonitrile at a flow rate of 1.0 ml/min. The column used was an Ultimate SHISEIDO PAK C18 ACR (4.6 mm × 250 mm, 5 μm), and the detector wavelength was set to 203 nm. The samples were performed in triplicate for each experiment. The cumulative release mass and percentage at the intervals of each time point was calculated.

Additional information and details regarding the *in vitro* activity studies are available in the Materials and Methods section in the Supporting Information.

### 2.5 *In Vivo* Wound Healing Studies

#### 2.5.1 Animal Experimental Protocol

All animal experiments were conducted in accordance with the relevant laws and institutional guidelines, and were approved by the Animal Ethics Committee of Jilin Agricultural University. Thirty-six healthy male ICR mice weighing 20–30 g were used in this study. The animals were divided into clean cages under a temperature-controlled condition of 23 ± 2°C. After being reared adaptively for 1 week, the mice were anesthetized *via* intraperitoneal injection of chloral hydrate (0.3 mg/kg body weight). Subsequently, the dorsal hair on their backs was shaved using an electric razor, before being depilated up to the depth of loose subcutaneous tissue and disinfected with 70% alcohol. Next, 10 mm diameter circular full-thickness wounds were created on the back of each mouse under aseptic conditions. These wound mice were randomly distributed to three groups (*n* = 12 mice per group): mice in group I did not receive any treatment (naked injury–Control), group II mice were treated with Blank-Gel, while mice in group III were once daily treated with Rg3-Gel, the total amount of each administration was 200 ml. The wound area of the lesions was photographed and measured (ImageJ software) at days 0, 4, 8, 12, and 16.

The degree of wound healing was expressed as the wound contraction ratio (WCR) to assess the wound-healing property. The WCR was calculated by the following equation:

Percentage of wound contraction (%)=(A0-At)/A0×100%,

where A0 and At represent the initial wound area and the wound area at certain times, respectively.

#### 2.5.2 Histopathological Evaluation

Histology was performed to evaluate the degree of healthy skin dermal organization, keratin staining, collagen staining, extracellular matrix composition and organization, and the structural integrity of cellularized constructs. Briefly, each mouse was euthanized at the experimental endpoint of 16 days. Wound lesion tissues were harvested and immediately cleaned with PBS, before fixing in 4% paraformaldehyde buffer for 48 h. The fixed samples were dehydrated in graded ethanol, cleaned with xylene, and embedded in paraffin blocks. Both the center and edge portions of the tissue were subsequently sliced into 5 μm sections and stained using hematoxylin–eosin (H&E) and Masson’s trichrome staining kits, respectively, according to the manufacturer’s guidelines. The slides were then imaged by light microscopy.

#### 2.5.3 Immunofluorescence Analysis

Using the same method described in the H&E staining section, the immunofluorescence of the wound section on the 16th day after surgery was conducted on sections with a thickness of 5 μm. After that, the tissue samples were deparaffinized with xylene, dehydrated with ethanol solutions (100, 95, and 80%), and blocked with PBS solution with 10% goat serum. The sections were further incubated with the primary antibody pan-keratin and then with specific secondary antibodies and DAPI. The stained sections were observed using a fluorescence microscope (Olympus IX71 Corporation, Tokyo, Japan), and the structures positive stained with pan-keratin (green) and DAPI nuclear staining (blue) were identified and photographed.

### 2.6 Western Blotting

Western blotting was conducted as described previously (Kim et al., 2018). The cell extracts were prepared using RIPA protein lysate (Beyotime, P0013) and measured by a Bio-Rad protein assay. The proteins were separated on 10% sodium dodecyl sulfate polyacrylamide gel electrophoresis (SDS-PAGE), blotted onto PVDF membranes, and the membranes were immediately blocked with 5% skim milk/bovine serum albumin (BSA) in tris-buffered saline and Tween 20 (TBST) for 1 h at room temperature, followed by incubation with primary antibodies at 4°C overnight. Following incubation, the blots were incubated with the corresponding horseradish peroxidase secondary antibody. The target proteins were visualized with an enhanced chemiluminescence system using ECL Advance Western Blotting Detection Reagents (GE Healthcare, Buckinghamshire, United Kingdom). Densitometric analysis for the quantification of the band intensities was performed using ImageJ software.

### 2.7 Analysis of Bacterial Inactivation in Wounds by DNA Sequencing

On day 16, bacterial samples from the wounds were collected by gently wiping the wounds of mice in the Control and Rg3-Gel groups back-and-forth with wet cotton swabs. Then, the swab tip was broken off and loaded into a provided sterile sample collection tube. The bacterial samples were shipped to Shanghai Personal Biotechnology Co., Ltd (Shanghai, China) for high-throughput sequencing. The paired-end raw sequencing data of the skin microbiota were de-multiplexed, joined, filtered, analyzed, and visualized using the Quantitative Insights into Microbial Ecology (QIIME2) pipeline. The obtained sequence information was used for flora composition analysis to systematically explore whether and how Rg3-Gel treatment affects the type and relative abundance of bacteria in the wound and to allow assessment of bacterial involvement in the wound after Rg3 treatment.

### 2.8 Statistical Analysis

Data are presented as the mean value ±standard deviation (SD). Graphs and statistical analyzes were performed by using GraphPad Prism 8 (GraphPad, United States) and SPSS 17.0 (IBM Corporation, United States). The differences among groups were measured by one-way ANOVA with Tukey’s HSD test *post hoc* comparisons. Statistical significance was defined as *p*-values < 0.05.

## 3 Results and Discussion

### 3.1 Synthesis and Characterization of Rg3-Gel

At present, together with the functional hydrogel, the utility of other active pharmaceutical ingredients (such as cells, antibacterial agents, growth factors, drugs, or proteins) produce extremely effective hydrogel dressings for use in wound healing (Cheng et al., 2020). Notably, a recent study exhibited a microsphere/hydrogel composite constructed from ciprofloxacin (Cip)-loaded poly (lactic-co-glycolic acid) (PLGA) microspheres and ginsenoside Rh2 showed excellent potential applications in topical treatment of skin infection (Sun et al., 2020). Furthermore, to prevail the limitations related to ginsenosides delivery, a therapeutic delivery system based on microemulsion composite hydrogel has been designed to improve the low water solubility of ginsenosides (Kim et al., 2018). Inspired by this, we presented polymeric materials and anionic surfactants based composite thermo-sensitive hydrogel system for delivery of Rg3 as a drug release vehicle (Rg3-Gel) in this work.

**FIGURE 1:**
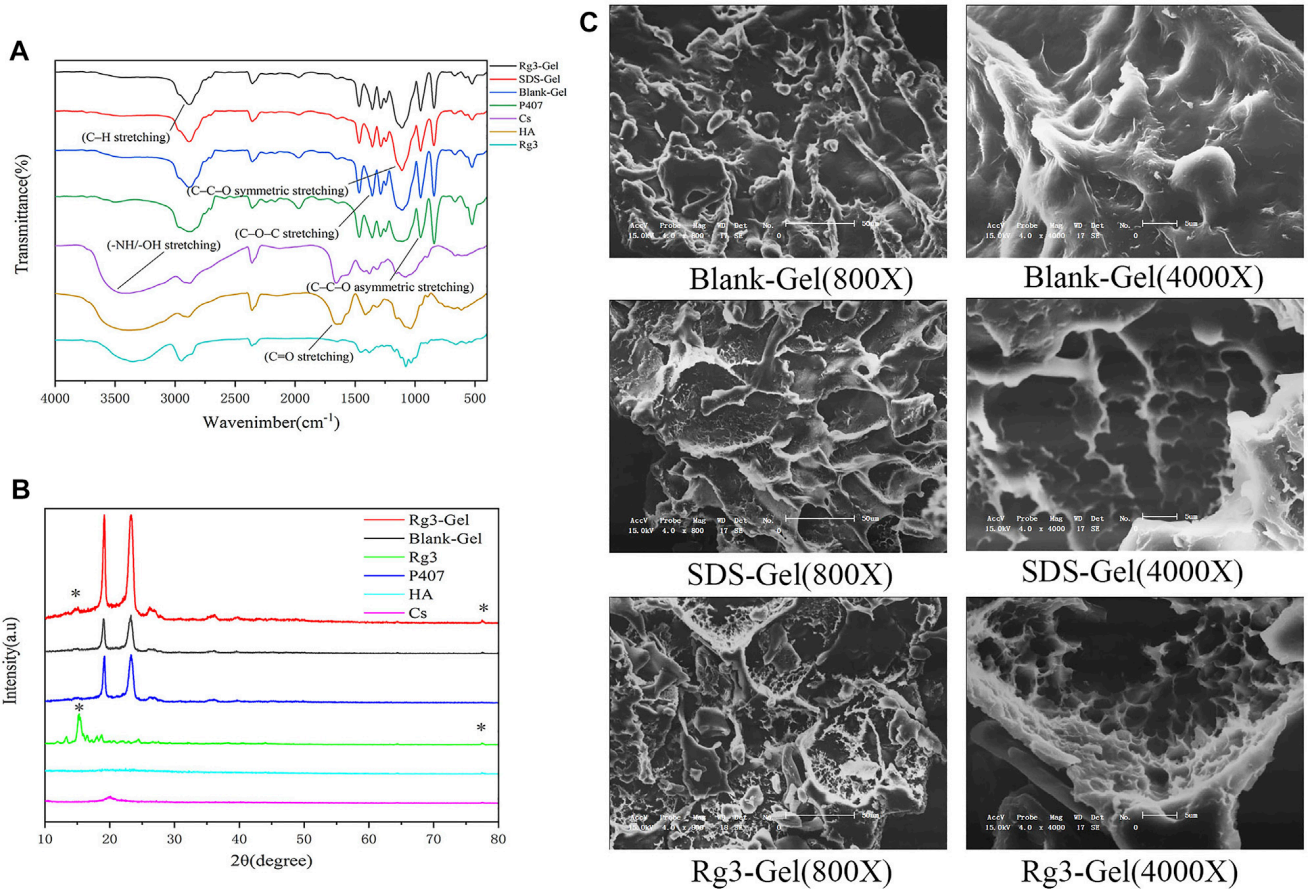
Structural characteristics of the Rg3-Gel. **(A)** Fourier transform infrared spectra of Rg3, neat P407, Cs, HA, Blank-Gel, SDS-Gel, and Rg3-Gel. **(B)** XRD pattern of Rg3, neat P407, Cs, HA, Blank-Gel, and Rg3-Gel. **(C)** SEM images of freeze-dried Blank-Gel, SDS-Gel, and Rg3-Gel samples (X800 & X4000).

The characteristic chemical bonding peak of Rg3-Gel is observed in Figure 1A. The hydrogel samples in the freeze-dried form showed (C–H stretching) and (C=O stretching) at 2,875 and 1,645 cm^−1^, respectively, and these peaks also included 1,282 cm^−1^ (C–O–C stretching), 1,110 cm^−1^ (C–C–O symmetric stretching), and 964 cm^−1^ (C–C–O asymmetric stretching). It was found that the chemical structure of Rg3 hydrogel was not only nearly consistent with that of the *in situ* hydrogel but also matched that of P407. All samples showed the disappearance of broad characteristic peaks at 3,200–3,600 cm^−1^ of the hydroxyl group (O-H or N-H stretching) vibration of the loaded drug compound. There is almost no chemical change in the combined spectrum of the Rg3 hydrogel compared to the blank hydrogel and P407, which is consistent with that reported previously for P407 as the main matrix (Soriano-Ruiz et al., 2020).

This result also appeared in the XRD pattern (Figure 1B), in which characteristic P407 diffraction peaks were clearly observed at 19.05° and 23.25°. The diffractogram of the drug encapsulated hydrogel and blank hydrogel also showed large diffraction peak patterns corresponding to their crystalline regions, as well as higher intensities. The diffraction sharp narrow peaks of the hydrogel encapsulated with Rg3 at 15.2° and 77.4° was due to the highly crystalline Rg3 structure. However, no change in the crystal phase due to chitosan doping was observed in the XRD spectra. This may be because the added chitosan is so small that it was below the XRD detection limit. The diffraction pattern may also indicate that Rg3 existed in the intrinsic crystal form in Rg3-Gel.

Simultaneously, the SEM of our hydrogel illustrated a significant difference structure in different hydrogel formulations (Figure 1C). The hydrogel formed after the addition of SDS showed a typical 3D porous morphology and appears looser and more porous in nature than the blank hydrogel. In addition, the Rg3 content did not significantly affect the pore structure. These SEM images indicated that the 3D structures of the hydrogel may contribute to the encapsulated and controlled release of drugs, as well as maintain a physiologically moist microenvironment and provide channels for gas exchange at the wound site to promote healing.

### 3.2 Properties of Rg3-Gel

**FIGURE 2:**
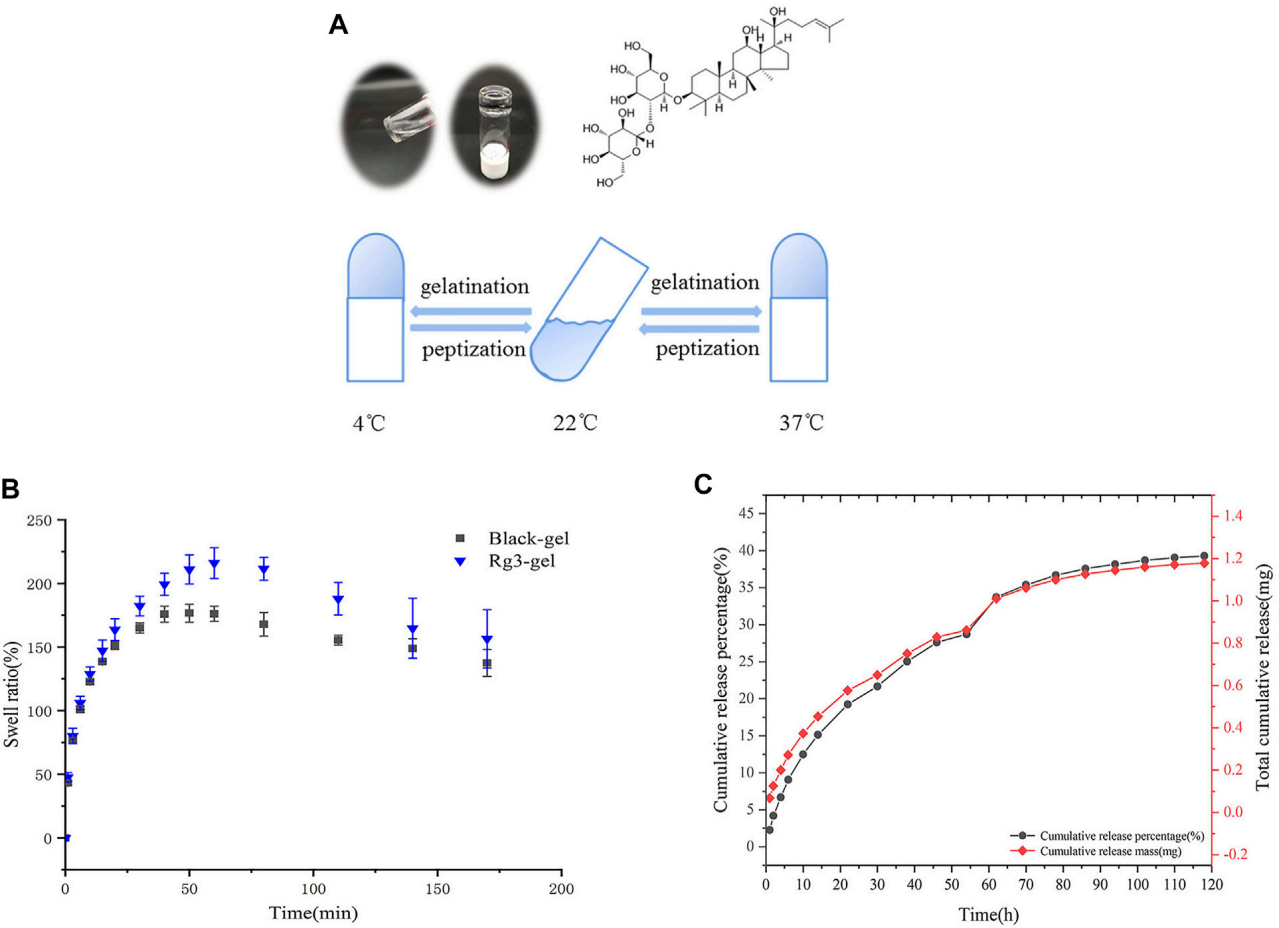
Excellent properties of Rg3-Gel. **(A)** Schematic and tube inversion images of Rg3-Gel with temperature (sol–gel transition). **(B)** Swelling characteristics of Blank-Gel and Rg3-Gel dry samples; data represent average of triplicate experiments ±SD (*n* = 3). **(C)** Cumulative release profile of Rg3 from the hydrogel sample at 37°C in 0.1 (M) PBS buffer by HPLC; data represent average of triplicate release experiments ±SD (*n* = 3). Antioxidant, antibacterial ability, and cytotoxicity of Rg3-Gel.

The relationship between the classical anionic surfactant sodium lauryl sulfate (SDS) with stable hydrogels was studied many years ago (Wu et al., 2012, 2017). Similarly, another report recommends that fibroin aggregation and gelation can be accelerated by SDS in a matter of minutes, based on hydrophobic interactions and electrostatic effects. The SDS-treated hydrogels are further reported to possess excellent cytocompatibility *in vitro* (Kao et al., 2020). The Rg3-Gel outlined in this study is transparent in appearance, and the diagram is shown in Figure 2A. Interestingly, we used the tube inversion method to record the gelation of the hydrogel, and found that the gelation formulation of the Rg3-Gel not only exhibited a phase transition at physiological temperature with the addition of SDS, but the hydrogel also solidified into semisolids (gels) at low temperature, which attests to it has bidirectional temperature sensitivity. Although the cause of its gelation process remains unknown, early studies reported that in addition to chemical reactions, physical interactions, such as hydrogen bonds, hydrophobic bonds, and electrostatic interactions, may occur during the gelation process (Wang X. et al., 2018).

Figure 2B shows the swelling properties of poloxamer-based Blank-Gel and Rg3-Gel to analyze the water uptake capacity of the hydrogels. Both hydrogels swelled rapidly within the first 25 min of immersion in PBS, and within 50 min of immersion, the swelling equilibrium was reached. However, Blank-Gel was equilibrated slower compared to the Rg3-Gel, probably due to the three-dimensional (3D) structure of the Rg3-Gel, which is looser and more porous. All of the results suggest that all hydrogels have abundant pores to take up large amounts of water; therefore, it can prevent the accumulation of exudates at the wound bed, making them suitable for wound dressing.

A good drug carrier can also enhance drug stability. HPLC chromatography was used to obtain the controlled Rg3 release process from the Rg3 hydrogel. The bioactive Rg3 was effectively encapsulated in the hydrogel and exhibited a representative long-term temperature-sensitive sustained release behavior. The Rg3 release profile is shown in Figure 2C. The Rg3 released from the Rg3 hydrogel showed a rapid burst release of approximately 9% over 6 h, and approximately 28.7% over 54 h. The sustained and controlled release of Rg3 could be observed, while the formation of a burst of Rg3 from the loose part of the hydrogel surface can promote early liberation. The drug cumulative release profile was consistent with its cumulative release percentage, and we detected a cumulative release of 1.18 mg of Rg3 over 120 h from 3 ml hydrogel. These Rg3 release behaviors of the hydrogels demonstrated their potential use as drug release carriers.

### 3.3 Antioxidant, Antibacterial Ability, and Cytotoxicity of Rg3-Gel

**FIGURE 3:**
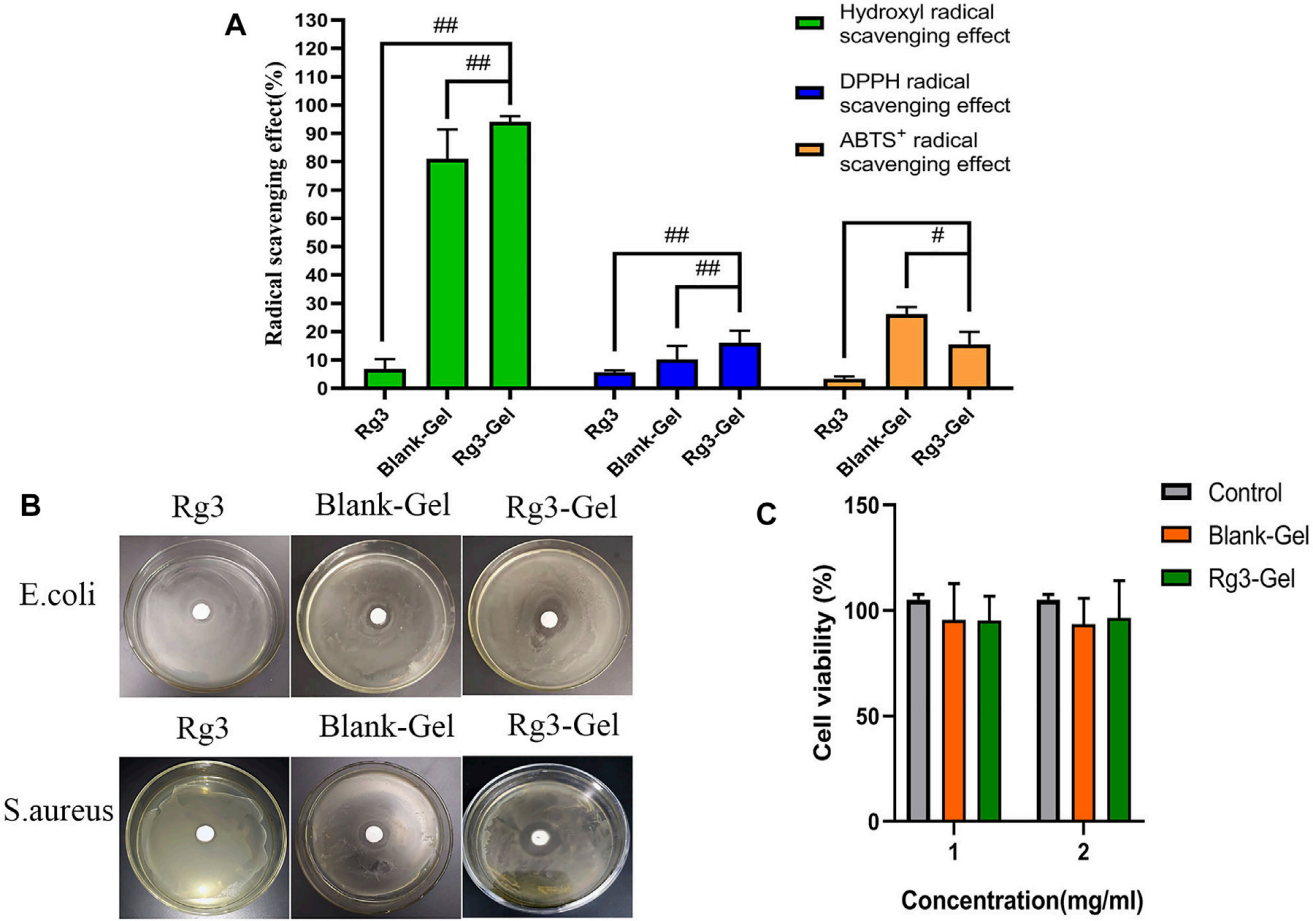
Antioxidant, antibacterial ability, and cytotoxicity of Rg3-Gel. **(A)** Each radical scavenging property of the Rg3, Blank-Gel, and Rg3-Gel (^#^ *p* < 0.05, ^##^ *p* < 0.01, and *n* = 3). **(B)** Antibacterial inhibition zones of the Rg3, Blank-Gel, and Rg3-Gel for *S. aureus* and *E. coli*, respectively (*n* = 3). **(C)** Cell viability of human HaCaT keratinocytes (MTT assay) treated with Rg3-loaded Rg3-Gel and Rg3-unloaded Blank-Gel samples; data represent average of five experiments ±SD (^#^ *p* < 0.05, ^##^ *p* < 0.01, and *n* = 5).

Free radicals are present at high levels in the wound site, and this results in oxidative stress and chronic inflammation, degeneration, and cell death, all of which compromise the wound-healing process. Rg3 has been reported to have excellent antioxidant properties and can scavenge free radicals (Kang et al., 2006). Here, the antioxidant activities of Rg3-Gel and pure Rg3 solution were evaluated by testing the removal efficiency of hydroxyl, DPPH, and ABTS oxide radicals. The results revealed a more obvious radical scavenging efficiency of the blank hydrogel combination (Figure 3A). The blank hydrogel combination showed 81.0, 10.3, and 26.27% scavenging of hydroxyl, DPPH, and ABTS, respectively, which were higher than single Rg3. The pure Rg3 solution showed no obvious scavenging efficiency at the same concentrations, while the hydrogel showed increased antioxidant capacity with the addition of Rg3 (except for the ABTS free radical scavenging efficiency). Taking the hydroxyl radical scavenging efficiency as an example, after adding Rg3 hydrogel, the hydroxyl absorption peak intensity was significantly reduced, indicating that these hydrogels have good antioxidant ability. These results indicate that the strong hydroxyl radical scavenging property blank hydrogel of the biopolymer combination could effectively combine with Rg3 to improve the antioxidant activity. Overall, Rg3-Gel has good antioxidant capacity and shows great potential as a wound-dressing material.

Bacterial infection is not only one of the main causes of wound infection but also one of the main factors inhibiting the wound-healing process. Thus, the desired wound dressing should have a good antibacterial activity to inhibit the propagation of microorganisms at the wound site and prevent the wound from being invaded by external bacteria (Chen et al., 2018; Song et al., 2019). In this regard, Figure 3B illustrates the antimicrobial activity of the hydrogel-loaded Rg3 compared to the hydrogel alone as the control sample. As has been observed, the prepared hydrogel, irrespective of whether or not it contained Rg3-loaded hydrogel, showed the effectiveness of inhibiting the growth of both bacterial species of *S. aureus* and *E. coli*. Furthermore, the mean diameters of the zone of inhibition recorded for Rg3-loaded hydrogel against *E. coli* and *S. aureus* were 20.3 ± 1.2 and 25.0 ± 1.1 mm, respectively, which were higher than those of Rg3 (*p* < 0.01) and the blank hydrogel (*p* < 0.01), revealing that Rg3-loaded hydrogels have greater antimicrobial potential. Similarly, the antibacterial activity of pure Rg3 was investigated against *S. aureus* and *E. coli*, the result of which is shown in Table 1. Statistical analyses revealed that pure Rg3 could combat those common pathogens associated with acute wounds. In addition, according to previous studies, chitosan exhibits good antibacterial toward bacteria (Gram-positive and Gram-negative), suggesting that the strong antibacterial activity of Rg3-Gel may be due to the synergistic effect of Rg3 and chitosan. Therefore, based on the aforementioned results, Rg3-Gel showed sufficient inhibition of bacterial growth to protect wounds.

**TABLE 1:** Bacterial inhibition zone of the different samples.

Sample name	Inhibition zone (mm)	
	*E. coli*	*S. aureus*
Rg3	15.2 ± 0.8	12.4 ± 0.7
Blank-Gel	14.2 ± 0.8	18.2 ± 0.9
Rg3-Gel	20.3 ± 1.2	25.0 ± 1.1

Diameter of inhibition zone of different samples. Data represent average of three experiments ±SD.

Excellent cytocompatibility is one of the most important properties of materials in biomedical applications. The cytotoxicity level of materials is generally divided into four level grades, where relative survival rates >70% (Grade 1) can be considered non-toxic (Zhang et al., 2021). Figure 3C shows the results of cell viability, following treatment with Blank-Gel and Rg3-Gel. The cell viability of two treatment groups was statistically significant (*p* < 0.05) compared to the controls. The relative cell survival rates of the two hydrogels at different concentrations were >70% after 24 h. The results prove that Rg3-Gel had no toxicity toward the treated samples, indicating that Rg3-Gel can provide an ideal environment for cell culture and may facilitate the rapid repair of skin cells.

### 3.4 Evaluation of Wound Healing

**FIGURE 4:**
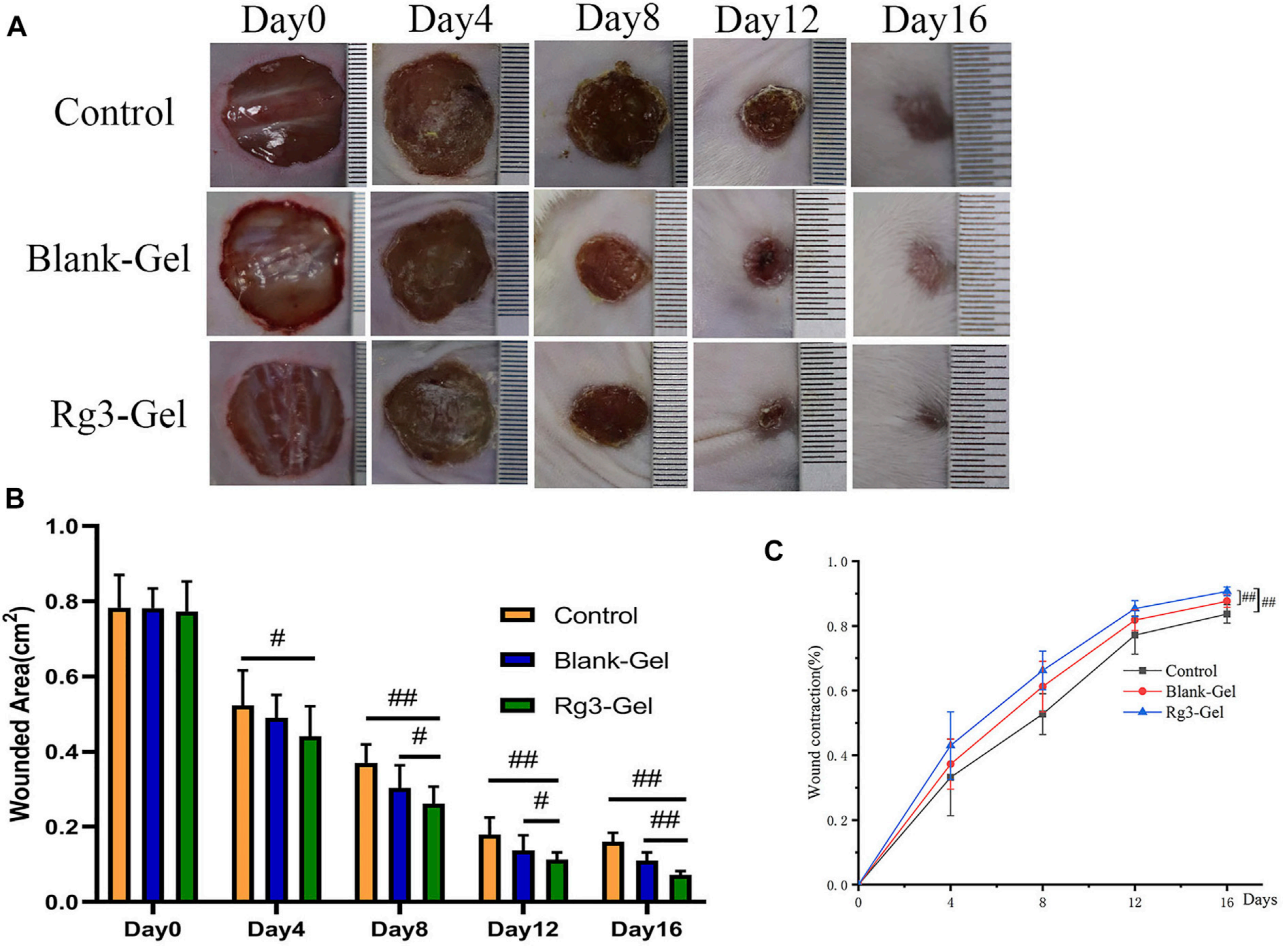
*In vivo* wound healing effect of Rg3-loaded Rg3-Gel on full-thickness excision wound model. **(A)** Wounds photographs at 0, 4, 8, 12, and 16 days post-injury in Control, Blank-Gel, and Rg3-Gel group. The scale bar indicates 10 mm. **(B)** Quantification analysis of wound areas at 0, 4, 8, 12, and 16 days post-injury in each group (#*p* < 0.05, ##*p* < 0.01, and *n* = 12). **(C)** Wound closure represented as the percentage of reduction of the initial area in each group during post-injury 14 days. Control, Blank-Gel, and Rg3-Gel represent the Control group, the blank hydrogel group, and the Rg3 hydrogel group, respectively (#*p* < 0.05, ##*p* < 0.01, and n = 12).

The faster the wound closes, the more effective the treatment. A full-thickness skin wound on the back of the mice was created and treated with Blank-Gel and Rg3-Gel. Representative images of the change in size of the wound areas from four groups on post-surgery days 0, 4, 8, 12, and 16 are exhibited (Figure 4A). On day 4 post treatment, reduction in wound size was observed in the animals to some extent, while Rg3-Gel exhibited the smallest wound area on days 8, 12, and 16 (*p* < 0.05 and *p* < 0.01). Moreover, the mice in the Rg3-Gel group showed a much higher reduction in the wound area as compared to the Blank-Gel (*p* < 0.05 and *p* < 0.01) (Figure 4B). Consistent with the gross observation, the Rg3-Gel group exhibited faster healing rates than those observed in the other groups during the whole healing process, with 90.68 ± 1.3% closure rates on day 16, while the wound-healing rates of the *in situ* hydrogel and the Control group were 87.63 and 83.67%, respectively (Figure 4C). This finding indicates that the hydrogel supports Rg3 sustained release and biological activity.

### 3.5 Histological and Angiogenesis Analyses

**FIGURE 5:**
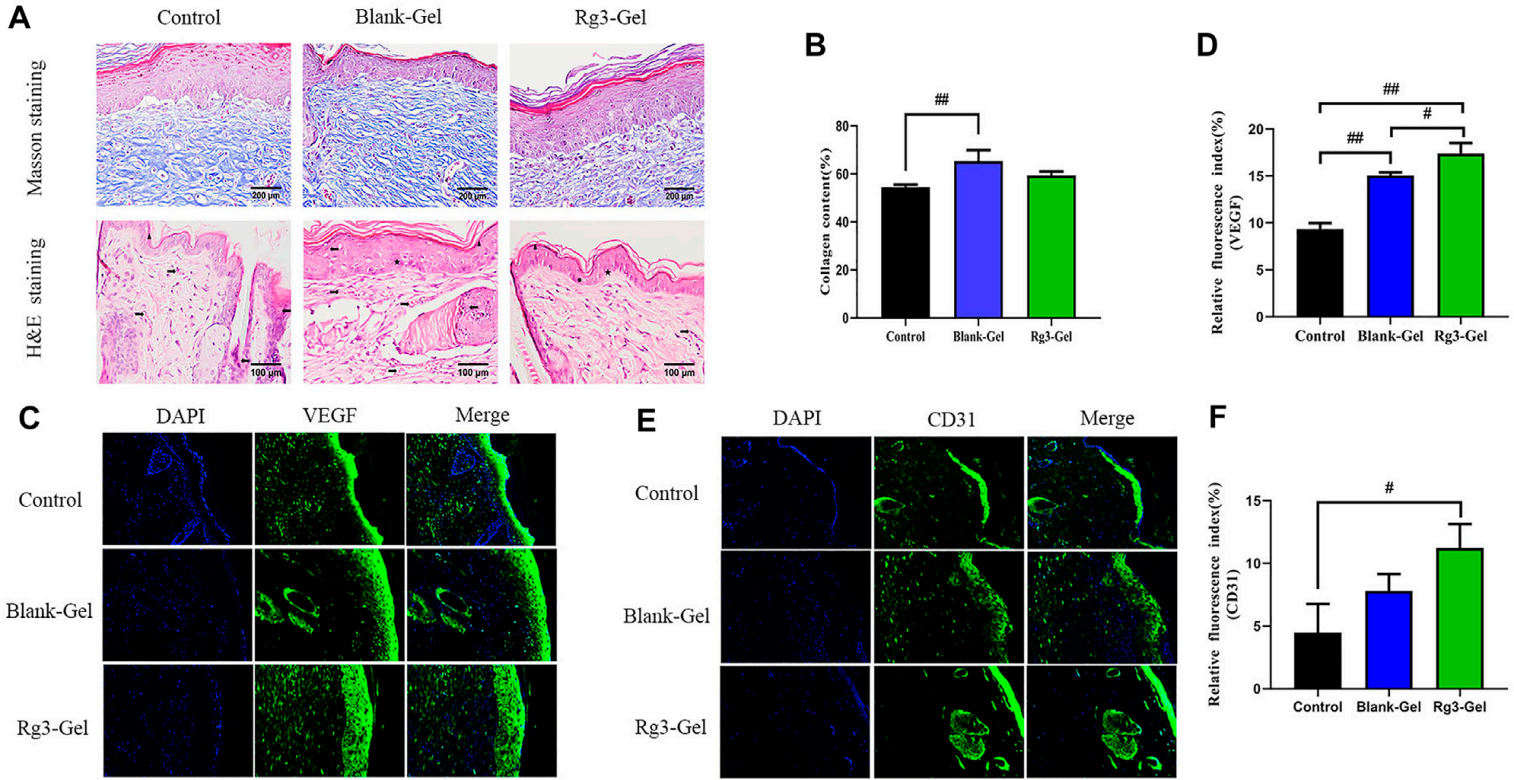
Histological and vascularization assessment after different treatments. **(A)** Masson trichrome staining and H&E histological staining of wounded skin tissue sections in different groups at 16 days post-wounding (×400 magnification and scale bar 200,100 μm). The right arrow represents capillary vessels, while the left arrows represent inflammatory cells. The triangle, star, and circle represent *epidermis*, hair follicles and glands, respectively. **(B)** Proportion of collagen fibers in the regenerative tissues (*n* = 3). **(C,D)** VEGF immunofluorescence staining images and quantitative analysis in wound areas of different groups. **(E,F)** CD31 immunofluorescence staining images and quantitative analysis in wound areas of different groups (n = 3).

**FIGURE 6:**
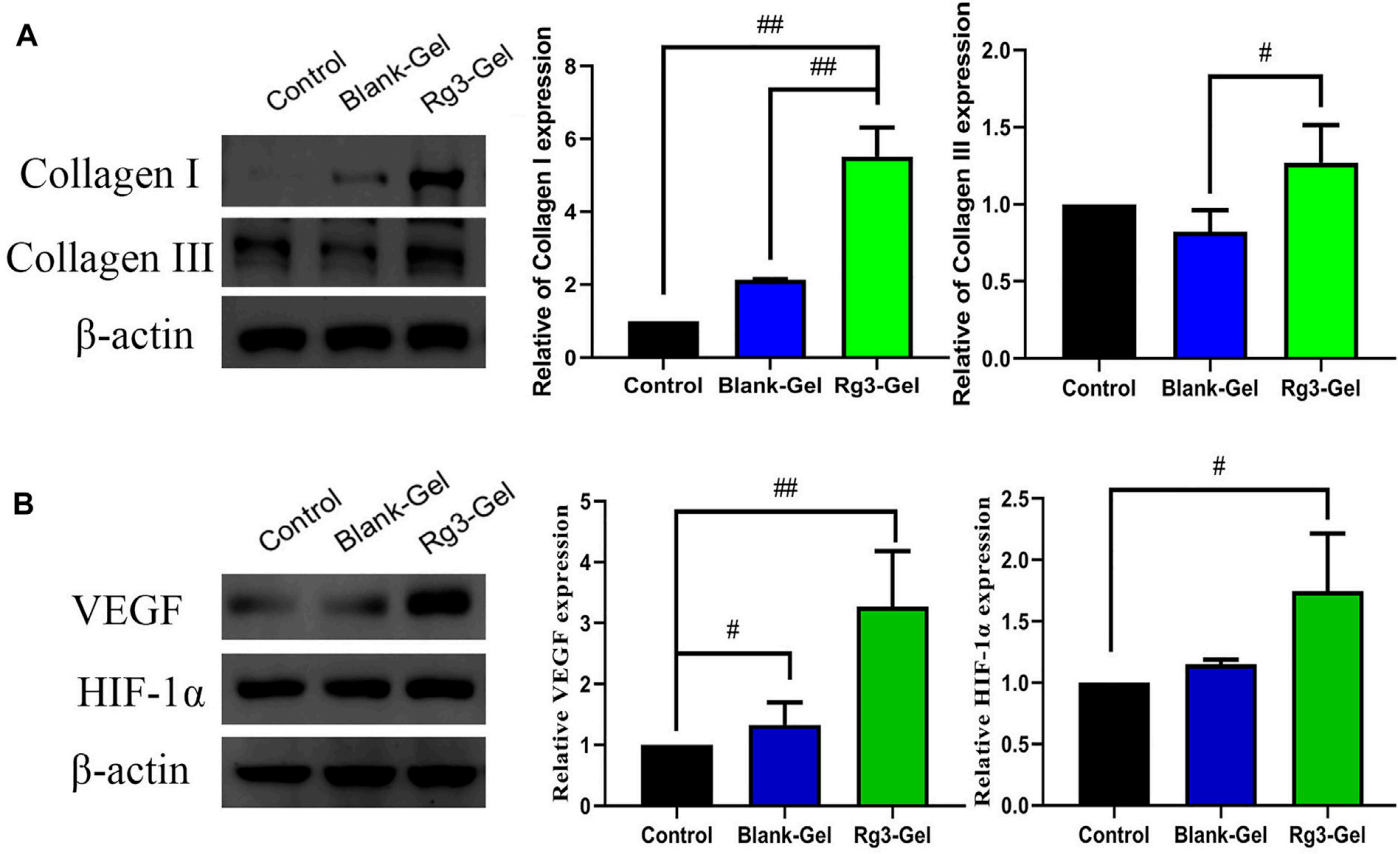
Effects of Rg3-Gel on collagen and angiogenesis at wound-healing sites. **(A)** Representative western blots and their quantitative analysis of Collagen I and Collagen III proteins in skin tissue of three groups. **(B)** Representative western blots and their quantitative analysis of VEGF and HIF-1α proteins in skin tissue of three groups (#*p* < 0.05, ##*p* < 0.01, and *n* = 3)

Collagen participates in the healing process through its role in cell migration and new tissue development. By Masson staining, collagens are stained blue and keratins are stained red. Masson trichrome staining revealed that all groups showed more collagen deposition compared to the Control group (Figure 5A). ImageJ software was used to calculate the collagen content of the wound dermis, represented by the average optical density (Figure 5B). As a result, the highest collagen deposition and a better collagen array were found in the Blank-Gel group, which increased in the mean area (%) of collagen deposition compared to other groups. These findings indicate that Blank-Gel accelerated the healing process by promoting ECM deposition at the wound site. Although the increased collagen content was weaker in the Rg3-Gel group than that in the Blank-Gel group, the collagen-based protein quantification results indicated that collagen I and III expressions under the wound increased significantly in the Rg3-Gel group compared to the Blank-Gel group (Figure 6A, *p* < 0.01 and *p* < 0.05). Collagen has been reported to stimulate the adhesion, proliferation, migration, and epidermal differentiation of human keratinocytes, as well as to promote skin regeneration and wound healing in rats (Kim et al., 2014; Chen et al., 2019). The altered collagen content in the Rg3-Gel–treated wounds could be attributed to the introduction of Rg3 into the hydrogel system to a large extent, which may promote collagen deposition and accelerate wound healing.

H&E staining confirmed the superiority of the Rg3-Gel group in terms of histopathological repair. The normal skin of mice is mainly composed of three layers: the *epidermis*, dermis, and hypodermis. According to the H&E staining results, the dermal layer was still collapsed in the Control group, while the Blank-Gel–treated wound showed the basic structure of the epithelial layer and an intact dermis layer, with no obvious edema, and a few hair follicles and glands (Figure 5C). However, the skin epithelium and appendage arrangement were not as regular as those in the wounds treated by Rg3-Gel. Our data showed that slightly fewer inflammatory cells and many skin appendages were observed in the Rg3-Gel group. The infiltration of inflammatory cells into the wound site occurs when the wound is infected by bacteria. Relative hypoxia is another characteristic feature of tissue growth or following tissue injury and impaired blood flow, and stimulating angiogenesis and restoring the vascular network can provide the required nutrients and oxygen for the wound repair process (Ahluwalia and Tarnawski, 2012; Feng et al., 2021). Indeed, it has long been known that angiogenesis plays an important role in multiple stages of the wound-healing process. In the current staining plot, the effect of Rg3-Gel on angiogenesis at the wound site is unclear.

The lack of nutrients and oxygen is the principal factor that stimulates angiogenesis. Angiogenesis is also the result of endothelial cell proliferation and the production of various growth factors and cytokines, which provide the necessary cellular and molecular signals for a normal healing process. Among them, vascular endothelial growth factor (VEGF) is the most important mediator in promoting angiogenesis (Ferrara et al., 2003; Li et al., 2003). To further explore the potential regulatory effect of Rg3-Gel on angiogenesis, we verified the formation of blood vessels by immunofluorescence staining with the angiogenesis markers platelet endothelial cell adhesion molecule-1 (CD31) and VEGF (Figures 5C–F). The results showed that Rg3-Gel significantly enhanced the expression of VEGF and CD31 in mouse skin wounds compared with the Control group, thereby promoting angiogenesis (*p* < 0.01 and *p* < 0.05). In addition, the VEGF protein detected by western blotting was highly expressed in the wounds of the Rg3-Gel group (Figure 6B, *p* < 0.01). We also noted that the hypoxia-inducible factor (HIF)-1α (HIF-1α) protein level were significantly increased in the Rg3-Gel group compared to the Control group (Figure 6B, *p* < 0.05). HIF-1α is a major transcription factor, which participates in VEGF-mediated angiogenesis under hypoxic conditions, as well as induces the expression of multiple critical molecules for wound healing (Rey and Semenza, 2010). The quality and quantity of neovascularization determine the quality of wound healing. Previous studies have reported the use of 20(S)-protopanaxadiol (PPD) in wound-healing therapy by stimulating angiogenesis through HIF-1α-mediated VEGF expression. Micromolar concentrations of Rg3 have been shown to increase nitric oxide (NO) production in the vascular endothelium, promote human endothelial cell proliferation, migration, and tube formation *in vitro*, and promote as *ex vivo* endothelial sprouting (Chen et al., 2010; Hien et al., 2010; Kwok et al., 2012). Taken together, these findings provide solid evidence that Rg3-Gel promotes angiogenesis. These results are consistent with our previous collagen expression results, suggesting that Rg3-Gel may enhance wound healing by promoting angiogenesis.

### 3.6 Immunofluorescence Analysis of Pan-Keratin

**FIGURE 7:**
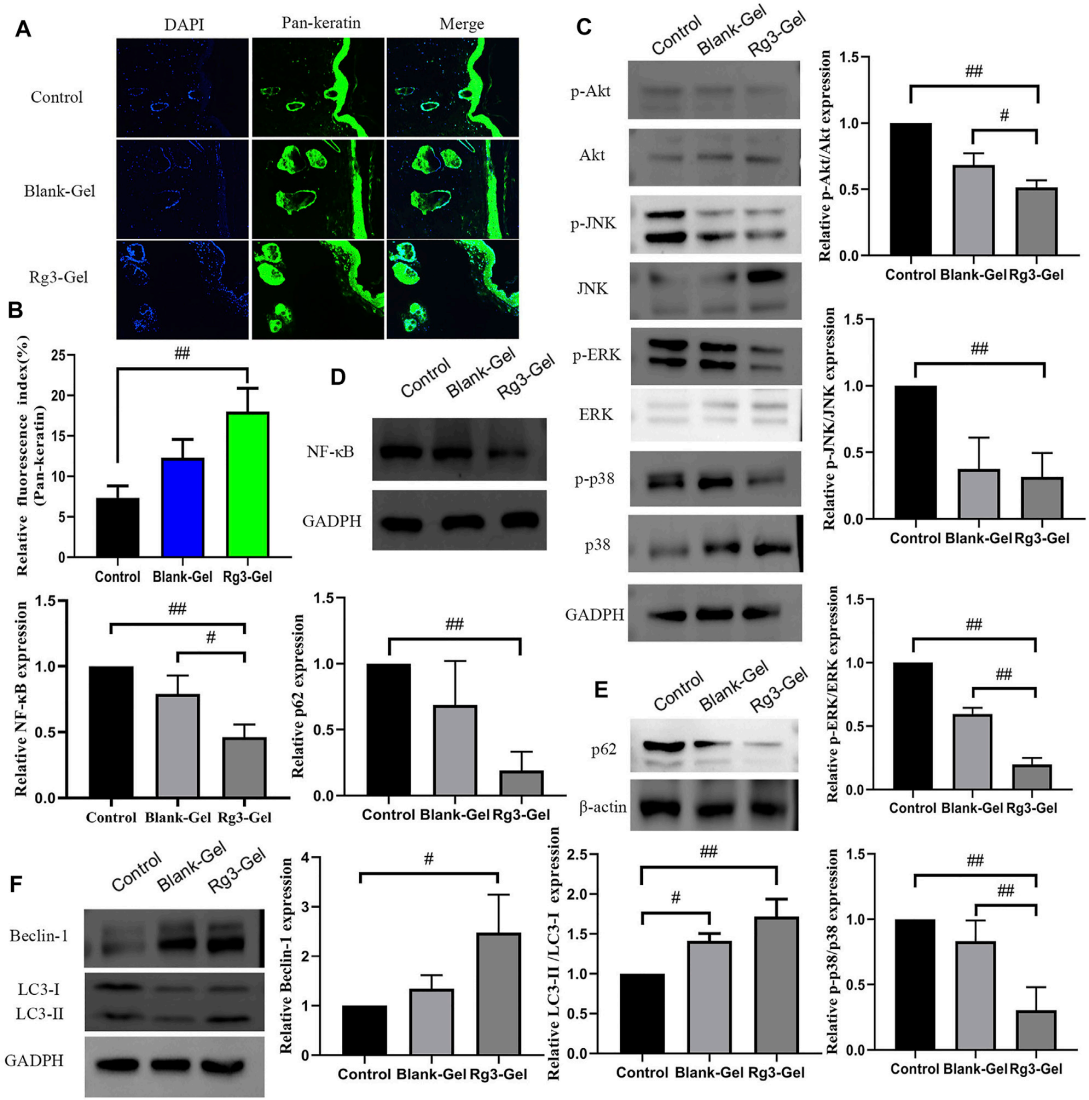
Effect of Rg3-Gel on the expression of various proteins in wound tissues. **(A)** Representative images of pan-keratin immunofluorescence staining on day 16 post surgery. **(B)** Expression of pan-keratin protein affected through the Rg3-Gel treatment in skin tissue in mouse (#*p* < 0.05, ##*p* < 0.01, and *n* = 3). **(C)** Representative western blotting of Akt, JNK, ERK, p38, and their phosphorylated proteins in skin tissue of three groups. **(D)** Effects of different treatment on the protein expressions of NF-κB, **(E)** p62, **(F)** Beclin-1, and LC3 (#*p* < 0.05, ##*p* < 0.01, and *n* = 3). Analysis of the MAPK/NF-κB signaling pathway and expression of autophagy-related proteins.

Keratins (KRT) are the largest subfamily of intermediate filaments (IFs) and the most abundant cellular proteins in simple epithelial cells. KRTs represent the main component of the epithelial cytoskeleton, which not only provides structural support to epithelial cells but also contributes to cell-type-specific functions, such as regulating cell proliferation, migration, adhesion, metabolism, and inflammatory features of keratinocytes (Zhang et al., 2019). Pan-keratin is a marker of re-epithelialization of the extracellular matrix. Previous studies on these Rg3 compounds have focused on their anticancer effects, with few reporting their effects on keratin (Sun et al., 2017). More recently, keratin has been reported in the use of novel biocompatible materials, such as membranes and hydrogels, in tissue engineering scaffolds, wound dressings, and surgical interventions, among others (Volkov and Cavaco-Paulo, 2016). Normal expression of keratin proteins is responsible for maintaining the structural stability and integrity of keratinocytes, and its mutation or abnormal expression is related to various skin diseases. In this study, immunofluorescence imaging of pan-keratin was conducted to assess the formation of a new KRT network. As demonstrated in Figures 7A,B, the expression of pan-keratin in the Rg3-gel group was significantly higher than that in the other groups, especially the Control group (*p* < 0.01). This result concluded that the enhancement of hydrogel system-mediated wound healing by Rg3 may occur through stimulation of pan-keratin.

### 3.7 Analysis of the MAPK/NF-κB Signaling Pathway and Expression of Autophagy-related 496 Proteins

The canonical transcription factor NF-κB is an accepted upstream trigger of the pro-inflammatory signal, which is activated by reactive oxygen species (ROS) and drives the expression of numerous genes, including those of pro-inflammatory cytokines, chemokines, adhesion molecules, and enzymes (Best et al., 2019). P65 is one of the important heterodimer members of the NF-κB family, the expression of which has been shown to be inhibited by Rg3 (Ma et al., 2020). Following activation by ROS, NF-κB can activate signaling pathways, including MAPKs and PI3K/Akt, which are crucial to regulating inflammation and producing inflammatory factors. The MAPKs regulate cell growth and differentiation as well as in the control of the cellular responses to cytokines and various stresses (Kwon et al., 2018; Best et al., 2019). To confirm the anti-inflammatory *in vivo* results, the effects of Rg3-Gel on the activation of MAPK family members, including p38 MAPK, JNK, ERK, and AKT (all of which are involved in the early stage of autophagy), were determined by western blotting. ROS are necessary participants in mitogen-activated protein kinase (MAPK) pathways, activated p38 and ERK signaling proteins are involved in the main signaling pathways for migration and proliferation of endothelial cells, and the MAPK signaling pathway is also responsible for the activation of nuclear factor-kB (NF-κB). Moreover, JNK is principally activated by ROS, but can also be activated by many other stimuli, including cytokines, hormones, cell stress, toxins, drugs, and metabolic changes (Shen et al., 2015; Niu et al., 2020). Inflammation occurs following wound formation as an adaptive response ubiquitous to both acute and chronic wounds. This inflammation induces and stimulates NF-κB and its upstream major translocation regulator, mitogen-activated protein kinases (MAPKs), including p38, ERK1/2, and JNK (Figure 7C). Of note, a substantial decrease in the ratio of phospho-Akt/Akt was detected following the treatment with Blank-Gel and Rg3-Gel (*p* < 0.01). Similar alterations in the ratio of phospho-JNK/JNK were also detected (*p* < 0.01), while treatment with specific inhibitors of ERK decreased the phosphorylation (*p* < 0.01). We detected low levels of p38 MAPK phosphorylation, an inhibitor of NF-kB, in the skin tissue of hydrogel-treated wounds (*p* < 0.01), while NF-κB was decreased to a similar level (*p* < 0.01, Figure 7D). Taken together, these findings show that the hydrogel loaded with Rg3 is apparently involved in inhibition of the inflammatory response in wound healing. This indicates that the use of hydrogel treatment in the wound can inhibit the activation of NF-κB and reduce inflammation, at least partially, by inhibiting the MAPK and NF-κB signaling pathways.

Autophagy is an essential homeostatic cellular process, which degrades damaged and dysfunctional organelles and proteins under highly conservative conditions (Sil et al., 2018). Several studies have proved that the NF-κB-p62 mitophagy pathway specifically inhibits pro-inflammatory processes to help restore homeostasis. The activation of autophagy significantly reduced the accumulation of p62, the expression of cathelicidin/LL-37, and the production of inflammatory cytokines. In short, NF-κB exerts an anti-inflammatory effect by delaying accumulation of the autophagy receptor p62/SQSTM1 (Lee et al., 2011). Further research indicates that autophagy is also related to the AMPK pathway, in which phosphorylated AMPK deactivates mTOR, which in turn activates the expression of LC3 and induces autophagy (Lu et al., 2021).

P62, one of several primary regulators of skin inflammation, was previously shown to have roles in the control of epidermal processes (Sukseree et al., 2021). P62 can modulate the degradation of ubiquitinated proteins during autophagy and acts independently as an adapter protein in autophagy. P62 not only induces cellular proliferation by activating the NF-κB pathway but also plays an important role in cell signaling crossroads, such as cellular apoptosis, inflammation, and autophagy (Usategui-Martín et al., 2020). To further elucidate the role of Rg3-Gel associated with the induction of autophagy *in vivo* under wound-healing conditions, the protein levels of downstream autophagy-related markers in the healed skin wounds were evaluated. We found an inverse correlation between p62 and Beclin-1 levels in skin tissue at wound healing sites of mice, whereas the highest p62 expression was present in the Control group (*p* < 0.01, Figure 7E). Several studies have proved that the JNK/Beclin-1 pathway plays a crucial role in mediating autophagic cell death. The downstream targets of JNK include the transcription factor c-Jun, which translocates to the nucleus after JNK-mediated phosphorylation. Other reports showed that the association between JNK and c-Jun not only activates c-Jun but also stabilizes c-Jun. When activated, the JNK-mediated phosphorylation of both Ser63 and Ser73 at the c-Jun N-terminal activates c-Jun and potentiates its transcriptional activity, thereby enhancing the transcriptional activity of Beclin-1. This process is important for the formation of autophagosomes and mediates the localization of other autophagy proteins on the autophagy precursor membrane (Sheng et al., 2021). The literature suggests that autophagy is impaired in the diabetic skin model due to the decreased expression of beclin-1 and LC3-II (Chowdhury et al., 2019). In line with this observation, as shown in Figure 7F, expression levels of Beclin-1 protein were lowest in Control group mice and highest in Rg3 hydrogel mice (*p* < 0.05). Similarly, low levels of Beclin-1 expression were found to be inversely correlated with p62 and positively correlated with LC3 in this study. Compared to the Control group, Rg3-Gel intervention significantly upregulated the expression of LC3 proteins (*p* < 0.01). The protein levels of these markers were upregulated by hydrogel treatment, and this effect was more pronounced with Rg3-Gel than Blank-Gel treatment, although no statistical differences existed between the Control and treated groups. From these findings, we concluded that Rg3-Gel stimulates autophagy *via* Beclin-1 and LC3 to significantly accelerate wound healing.

### 3.8 Analysis of Wound Bacterial Effect by DNA Sequencing

**FIGURE 8:**
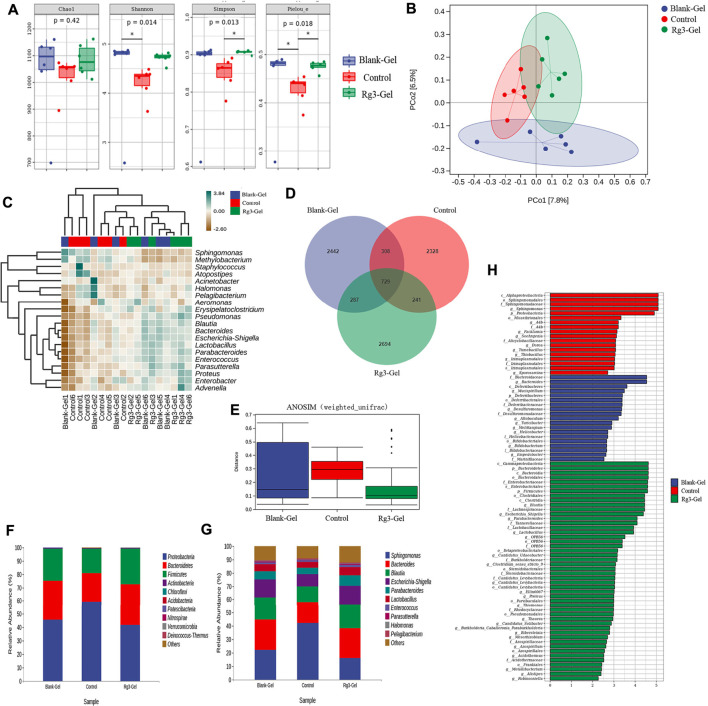
Effects of different treatments on wound flora. **(A)** Box plots of four different alpha diversity measures. **(B)** Multivariate analysis of beta-diversity: PCoA results based on weighted UniFrac (consider sequencing amounts when analyzing) across the three different sample groups. Blue, red, and green colors represent samples from Blank-Gel, Control, and Rg3-Gel individuals, respectively. **(C)** Correlation heatmap between three groups important differential wound microbes and the top 20 genera in abundance. **(D)** Venn diagram of shared and unique bacterial genera in the skin wound microbiome. **(E)** One way ANOSIM analysis (test statistic R > 0). **(F) (G)** Histogram of species distribution was used to compare the differences of relative abundance in phylum and genus levels between different groups. **(H)** LDA chart. The score was obtained by LDA analysis (linear regression analysis) (*n* = 6).

Microbiota induces a form of adaptive immunity that couples antimicrobial function with tissue repair. The purpose of microbiota manipulation in skin wound healing is to prevent pathogen infection and increase the proportion of beneficial microbiota. Numerous studies have investigated the microbial differences between healthy skin and diseased skin to find a cure to restore health. It is unclear what alterations in the microbiome are caused by the Rg3 hydrogel or whether it will improve the imbalance of the microbiota and contribute to skin wound healing. Generally, there exist four prevailing skin-associated taxonomic units on the skin: Actinobacteria, Proteobacteria, Firmicutes, and Bacteroidetes, all of which are actively involved in the infection and repair of skin wounds. It has been reported that certain skin bacteria can convert aromatic amino acids (AAA) into trace amines (TA), which accelerate wound healing, further confirming the significance of the epidermal microorganisms for wound healing (Luqman et al., 2020). Metagenomics is a novel method to detect species, population structure, diversity, and evolutionary relationships of microbiomes by high-throughput sequencing technology. In this study, metagenomics technology was used to detect the wounds of mice treated with hydrogel and to explore the microbial changes in the wounds after the introduction of Rg3. These models are displayed in Figure 8.

We analyzed the microbial communities on the wound surface of each group of mice using 16S rRNA Illumina high-throughput sequencing. The Wayne diagram in Figure 8D shows that the Blank-Gel, Control, and Rg3-Gel groups contained 3,766, 3,606, and 3,951 bacterial operational taxonomic units (OTU), respectively. The Rg3-Gel group had the most abundant microbes and the control the least, which indicates that the microbial diversity of untreated in wound is reduced compared to that of the Rg3 hydrogel treatment. Moreover, there was a trend toward increased species abundance and diversity of the two hydrogels compared to those observed in the Control group, as calculated by the alpha diversity metrics (Figure 8A). The differences within the sample groups were statistically significant (*p* < 0.05) for all diversity indices, including the Shannon, Simpson, and Pieloue indices. The Shannon diversity index is a measure of both species richness and the evenness of each species. A low index indicates low diversity, which is usually present in infections because a single microorganism dominates the balance and leads to disease, while a high index indicates greater diversity and is usually observed in normal, stable, and healthy communities (Kim et al., 2019). Evidence suggests that the imbalance of the microbial flora without any invading pathogens is an underlying cause of skin diseases. Therefore, simply eliminating all microorganisms is not a reasonable method to promote healing (Pang et al., 2020). The maintenance of skin health not only requires the inhibition of pathogenic bacteria but also to promote the growth of symbiotic bacteria (Grice et al., 2009). The bacterial diversity in the Rg3 hydrogel treatment group was significantly higher than that of the Control and blank hydrogel treatment groups; that is, mice that appeared to be more beneficial to assist wound healing by using Rg3-Gel to control microbial diversity. One-way ANOSIM analysis (test statistic R > 0) revealed significant differences between the three groups (Blank-Gel, Control, and Rg3-Gel) in terms of wound healing (Figure 8E), and the microbiota similarity of the samples can be observed by the clustering in the principal coordinate analysis plots (PCoA) (Figure 8B). Beta diversity analysis using the Jaccard similarity index and PCoA generation revealed a clear distinction in wound microbiota composition among the three groups, and the differences between the three groups of our study have statistical significance. According to reports, a continuous change of microbial diversity and community may indicate a tendency for wound tends to heal, while stabilizing in low diversity suggests that the wound may be chronic or not conducive to healing (Pang et al., 2020). Rg3-Gel treatments are consistent with the previously reported samples of normal skin, indicating a stable microbiota that returns to a “normal” state, following Rg3-Gel treatment during the wound-healing process.

**FIGURE 9:**
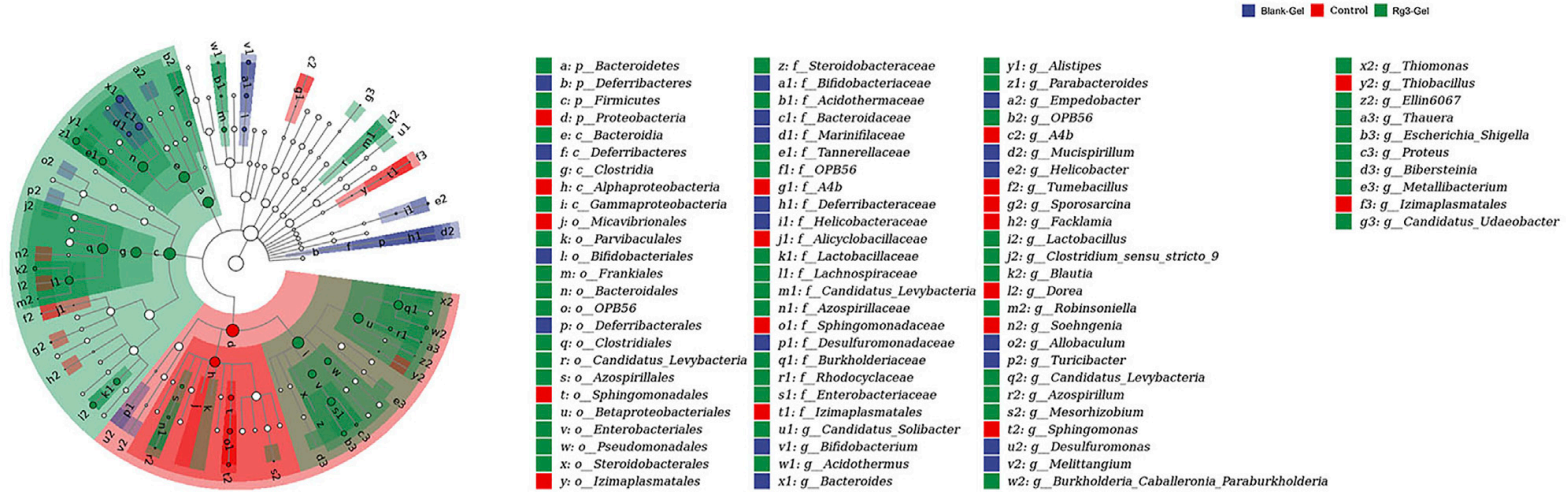
LEfSe cladograms analysis of wound surface taxa at genus levels among the different groups.

In addition, phylum- and genus-level taxonomic classifications of the wound microbiome revealed a variation in representative organism abundances between the two hydrogel treatment groups versus the Control group mice. In the current study, Proteobacteria, Bacteroidetes, Firmicutes, and Actinobacteria were observed as the four dominant microorganisms in the wound samples (Figure 8F). The relative frequency of *Proteobacteria* in the Rg3-Gel treatment group was reduced compared to that in the Control group, whereas *Bacteroidetes* and *Firmicutes* were increased. At the genus level, the six predominant groups identified were *Sphingomonas*, *Bacteroides*, *Blautia*, *Escherichia-Shigella*, *Parabacteroides*, and *Lactobacillus* (Figure 8G). The relative frequency of *Sphingomonas* was reduced in the two hydrogel treatment groups, while the other genera were increased. Moreover, the microbial community composition of these genera of the Rg3-Gel-treated mice varied more than that of the blank hydrogel. The heatmaps simultaneously illustrated *Sphingomonas*, *Methylobacterium*, *Staphylococcus*, *Atopostipes*, *Acinetobacter*, *Halomonas*, *Pelagibacterium*, and *Aeromonas* were the most prominent genera (Figure 8C). Moreover, *Bacteroides* normally function as friendly commensal bacteria in the host, and in rats with atopic dermatitis (AD) induction, the same treatments significantly decreased the relative abundance of the phylum *Bacteroidetes* and the genus *Bacteroides*. The intestinal microbiota seems to affect the health of the skin. Indeed, *Escherichia*/*Shigella* or *Veillonella* are found to be more prominent in the stools of patients with AD (Łoś-Rycharska et al., 2021). We observed a recovery in the level of *Lactobacillus* of skin microbiomes compared to the Control group mice skin, which showed that Rg3 hydrogel could regulate the skin microbiota at the genus level, similar to the findings of a previous study of diabetic skin (Jiao et al., 2020). *Sphingomonas*, a member of the phylum *Proteobacteria*, is an abundant genus that is identified on allergic skin. Many authors have indicated that the wound microbiome species, such as *Streptotrophomonas*, *Brachyrhizobium*, *Sphingomonas*, and *Phyllobacterium*, have a negative impact on wound healing, while *Leuconostoc*, *Enterococcus*, and *Bacillus* are beneficial to wounds (Mei et al., 2020). LEfSe (Linear discriminant analysis Effect Size) analysis showed the evolutionary relationship of different flora among groups (Figure 9). The dominant bacteria of the Control group were from *Proteobacteria*, while the representative bacteria of the Rg3-Gel groups expanded to *Bacteroidetes* and *Firmicutes*. Based on the results of linear discriminant analysis (LDA) (Figure 8H), Blank-Gel mice were characterized by a higher relative abundance of *Bacteroides*, *Mucispirillum*, *Desulfuromonas*, *Allobaculum*, *Turicibacter*, *Melittangium*, *Helicobacter*, *Bifidobacterium*, and *Empedobacter*, but lower amounts of *Sphingomonas*, A4b, *Facklamia*, *Soehngenia*, *Dorea*, *Tumebacillus*, and *Thiobacillus* than the Control group. In the Rg3-Gel treatment group, the main taxa with high LDA scores included *Blautia*, *Escherichia–Shigella*, *Parabacteroides*, *Lactobacillus*, *Clotridium_sensu_stricto*, and *Alistipes*. Culture-based studies of skin microbiota suggest that skin microbes can affect skin properties, immune responses, pathogen growth, and wound healing. Studies in recent decades have found that skin damage results in colonization by microorganisms, thereby increasing the risk of inflammation, which is reported to make damaged skin more susceptible to infection and ultimately affect wound healing. Overall, a shift in the microbiota composition by Rg3-Gel had a positive effect on wound healing. However, more studies must address the causal relationship between natural active ingredients and their influence on the composition of skin wound microbiota.

## 4 Conclusion

In this work, a new method for producing a temperature-sensitive injectable hydrogel containing Rg3 was developed using SDS as the gelling agent. Considering both safety and system simplicity, the hydrogels based on the chemical structure and temperature-sensitive properties of poloxamer were selected to use hyaluronic acid and chitosan along with Rg3 to accelerate the wound-healing process. The hydrogels, which show a unique porous morphology, promising biocompatibility, controlled Rg3 release, and good antioxidant and antibacterial activities, are ideal for use in wound healing. The experimental *in vivo* wound closure data show that the temperature-sensitive hydrogels with Rg3 could enhance wound contraction, re-epithelialization, collagen deposition, and angiogenesis, all of which accelerated the wound-healing process. The increase in autophagy protein expression suggests that hydrogels with Rg3 could enhance autophagy in wounds to promote healing. High-throughput data analysis showed that the biomimetic hydrogel with Rg3 increased the microbial diversity on the skin wound surface and reduced the abundance of harmful bacteria. Overall, the temperature-sensitive injectable hydrogels with Rg3 hold great promise for use in wound-healing applications.

## Data Availability

The original contributions presented in the study are included in the article/Supplementary Material; further inquiries can be directed to the corresponding authors.
