# Supplementary material for: Poloxamer 407 and Hyaluronic Acid Thermosensitive Hydrogel-Encapsulated Ginsenoside Rg3 to Promote Skin Wound Healing
Source: Front Bioeng Biotechnol. 2022 Jul 5;10:831007. doi: 10.3389/fbioe.2022.831007 (PMC9294355; doi:10.3389/fbioe.2022.831007)
Supplement: Supplementary file 1 [file DataSheet1.docx]

**Supplementary materials**





**Figure. S1. Calibration curve of** **(20S)-ginsenoside Rg3 as a function of its concentration.**

**

**

**Figure. S2. Calibration curve of** **(20R)-ginsenoside Rg3 as a function of its concentration.**
